# Threshold Responses of Bird Communities to Human Footprint: Testing the Intermediate Disturbance Hypothesis and Implications for Biodiversity Conservation

**DOI:** 10.1002/ece3.72683

**Published:** 2025-12-16

**Authors:** Xi Yang, Lishi Zhang, Piotr Tryjanowski, Frédéric Jiguet, Zheng Han, Haitao Wang

**Affiliations:** ^1^ College of Agricultural Hulunbuir University Hulunbuir China; ^2^ Animal's Scientific and Technological Institute, Agricultural University of Jilin Changchun China; ^3^ Department of Zoology Poznań University of Life Sciences Poznan Poland; ^4^ CESCO, UMR7204 MNHN‐CNRS‐Sorbonne Université Paris France; ^5^ School of Life Sciences Northeast Normal University Changchun China; ^6^ Jilin Provincial Key Laboratory of Animal Resource Conservation and Utilization, Northeast Normal University Changchun China; ^7^ Jilin Engineering Laboratory for Avian Ecology and Conservation Genetics, Northeast Normal University Changchun China

**Keywords:** diversity, human pressure, intermediate disturbance hypothesis, steppe birds, threshold

## Abstract

Human activities have profoundly altered natural ecosystems, driving widespread biodiversity declines. Birds serve as key environmental health indicators and exhibit high sensitivity to such changes. The Human Footprint Index (HFP) quantifies cumulative anthropogenic pressure, providing a robust framework to assess ecological responses to disturbance. We quantified species‐specific and community‐level thresholds in bird communities along an HFP gradient in northeastern Inner Mongolia using Threshold Indicator Taxa Analysis (TITAN). Using piecewise regression, we analyzed HFP‐driven changes in species richness and Shannon diversity to test the Intermediate Disturbance Hypothesis (IDH)—predicting peak biodiversity at intermediate disturbance levels. Our results reveal a community threshold at HFP around 14, indicating a shift in community composition, with species‐specific thresholds ranging from 3.37 to 43.22. Species richness and Shannon diversity peak at intermediate HFP levels (around 23.5), supporting the IDH, but decline at higher levels. These findings highlight the complex interplay between human impact and biodiversity, emphasizing the need for conservation strategies that consider both community composition and overall diversity, whereas addressing potential extinction debts and the roles of species traits in vulnerability.

## Graphical abstract

Human activities, quantified by the Human Footprint Index (HFP), drive biodiversity declines, with birds serving as sensitive indicators of ecosystem health. In northeastern Inner Mongolia, Threshold Indicator Taxa Analysis (TITAN) identified a community composition shift at HFP ≈ 14, with species‐specific thresholds ranging from 3.37 to 43.22. Piecewise regression supported the Intermediate Disturbance Hypothesis, showing peaks in species richness and Shannon diversity at intermediate HFP levels (≈23.5) before declines at higher intensities, underscoring the need for tailored conservation addressing both composition and diversity.

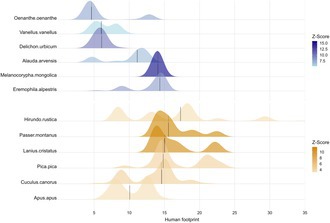

## 1 Introduction

Human activities have profoundly altered natural ecosystems, leading to widespread biodiversity loss and potential long‐term consequences such as extinction debts, where species persist temporarily but eventually decline because of lagged responses to habitat degradation (Hylander and Ehrlén 2013; Corlett 2015). The Human Footprint Index (HFP), a globally standardized metric ranging from 0 (minimal human influence, e.g., pristine wilderness) to 50 (maximum human pressure, e.g., dense urban areas with high infrastructure and population), integrates various anthropogenic pressures, including built environments, population density, agriculture, transportation infrastructure, and nighttime lights (Venter et al. 2016). This index provides a quantitative assessment of human influence on terrestrial ecosystems. High HFP values are consistently associated with significant biodiversity declines across multiple scales and taxa. Research shows that HFP negatively impacts species richness, abundance, and genetic diversity, especially for habitat specialists and large vertebrates (Tucker et al. 2018; Allan et al. 2019). The mechanisms connecting HFP to biodiversity loss are multifaceted. Land conversion for agriculture and settlements directly eliminates native habitats, whereas infrastructure like roads creates barriers to movement, isolates populations, and increases mortality through collisions and human‐wildlife conflicts (Ibisch et al. 2016). Areas with high HFP also face intensified resource extraction, such as logging or mining, and increased pollution—chemical, light, and noise—which stress wildlife and disrupt processes like pollination and predation (Kight and Swaddle 2011). Even moderate HFP levels can harm biodiversity, suggesting biodiversity degradation occurs well before landscapes are fully converted (Di Marco et al. 2017). Therefore, the HFP helps identify critical areas where reducing human pressure is urgent to protect biodiversity, particularly in contexts where extinction debts may mask immediate risks.

Species exhibit non‐linear responses to environmental gradients, including anthropogenic pressures. A “response threshold” refers to the point along a gradient—such as increasing habitat loss, pollution, or HFP—at which a species undergoes a significant change in its occurrence, abundance, or fitness, often leading to an abrupt decline towards local extinction (Huggett 2005; Groffman et al. 2006). Identifying these thresholds is vital for conservation, as they represent points beyond which populations may collapse rapidly, making recovery difficult. Different species exhibit varying sensitivities, with habitat specialists and K‐selected species (slow reproduction, long‐lived) generally having lower thresholds for disturbances like habitat fragmentation compared to generalists or R‐selected species (Swift and Hannon 2010). Ecological traits, such as nesting habits (e.g., ground‐nesting) or habitat preferences (e.g., open grasslands), may further influence vulnerability, as ground‐nesters are often more susceptible to trampling or predation in disturbed areas.

The Intermediate Disturbance Hypothesis (IDH), proposed by Connell (1978), suggests that local species diversity is highest at intermediate levels or frequencies of disturbance, rather than at either low or high disturbance extremes. Under low disturbance, dominant species often exclude others, thereby reducing diversity. At high disturbance, only a few tolerant species survive. In contrast, intermediate disturbance creates a balance by removing competitive species and providing space for a broader range of species to colonize. This dynamic promotes coexistence and enhances species diversity in a community (Connell 1978; Huston and Huston 1994). The applicability of IDH to anthropogenic disturbances, however, is debated and context‐dependent. Although natural disturbances like fires or floods can sometimes foster diversity under intermediate regimes, human‐caused disturbances often differ fundamentally in type, scale, intensity, frequency, and predictability (Fox 2013). Anthropogenic disturbances, such as intensive agriculture, urbanization, or pervasive pollution, often exceed the tolerance thresholds of most native species, leading to diversity declines rather than the hump‐shaped pattern predicted by IDH (Murphy and Romanuk 2012). Furthermore, IDH mainly addresses species richness, but it may not reflect changes in species composition, functional diversity, or ecosystem stability. For instance, high diversity under intermediate disturbance may be driven by early‐successional or generalist species, at the expense of specialized species that require stable habitats. Despite critiques, recognizing response thresholds remains essential to inform critical habitat protection and restoration targets, especially in avoiding extinction debts.

Birds are sensitive to environmental changes, and their responses are highly species‐specific, reflecting differences in ecological traits. Ground‐nesting species and habitat specialists (e.g., larks, bustards) are particularly vulnerable. For example, the Great Bustard ( *Otis tarda* ), which requires large, undisturbed tracts of intact grassland for lekking and nesting, shows steep population declines with even moderate increases in human infrastructure and grazing pressure (Rocha et al. 2013; Végvári et al. 2016). In contrast, some generalist species like the corvids may initially tolerate or even benefit from low‐intensity disturbance because of increased foraging opportunities in disturbed patches (Han et al. 2025), although their persistence often masks broader community degradation. At the community level, increasing the human footprint significantly alters steppe bird assemblages. Studies consistently report declines in species richness and abundance along gradients of disturbance intensity, particularly beyond moderate levels of grazing or habitat conversion (Liang et al. 2019). Intensified grazing often reduces vegetation height and cover, crucial for nesting concealment and invertebrate prey abundance, whereas cultivation fragments native grasslands and eliminates breeding habitat entirely. Human Infrastructure frequently increases collision mortality, acts as a barrier, and facilitates predator access (Bernardino et al. 2018; Gauld et al. 2022). Additionally, human activities can increase predation pressure from synanthropic species and expose wildlife to pesticides and other pollutants, further stressing bird populations (Tassin de Montaigu and Goulson 2020; Rigal et al. 2023).

The vast steppes of Inner Mongolia, part of the Eurasian grassland biome, support unique avian communities adapted to open, semi‐arid conditions. Notable species include the Great Bustard, Lesser Kestrel ( *Falco naumanni* ), Mongolian Lark ( *Melanocorypha mongolica* ), and Asian Short‐toed Lark (*Alaudala cheleensis*). These birds show varying sensitivities to anthropogenic pressures, including livestock overgrazing, land conversion to cropland, infrastructure development (e.g., roads, power lines, and settlements), and resource extraction (Zhuo et al. 2021; Zhang et al. 2023). Although the sensitivity of Inner Mongolia's steppe avifauna to anthropogenic pressures is well‐documented, few studies have used the HFP gradient to define statistically significant thresholds for species collapse or community turnover in this ecosystem. Furthermore, empirical tests of the Intermediate Disturbance Hypothesis, using breakpoint analysis (e.g., segmented regression) along HFP gradients, are notably absent, especially in contexts where human impacts diverge significantly from natural disturbance regimes. More critically, the use of complementary methods like TITAN to detect compositional thresholds that may precede diversity loss remains unexplored in this context. This limitation hampers the ability to define clear, generalizable HFP targets for conservation planning. This study seeks to address these gaps by examining steppe bird communities across an HFP gradient in Inner Mongolia. The study uses Threshold Indicator Taxa Analysis (TITAN) to identify species‐specific and community‐level thresholds (Baker and King 2010), and segmented regression to model responses in species richness and Shannon diversity. We hypothesize that: (1) Bird diversity (species richness and Shannon diversity) will peak at intermediate HFP levels, reflecting a balance between species sensitive to human disturbance and those that are more tolerant or benefit from it. (2) The community threshold, signaling a shift in species composition, will occur at a lower HFP level than the diversity thresholds, indicating that changes in community structure precede declines in overall biodiversity. By identifying these thresholds and testing the hypotheses, this study aims to improve our understanding of how human impacts affect avian biodiversity and provide actionable insights for conservation planning.

## 2 Materials and Methods

### 2.1 Study Area

**FIGURE 1:**
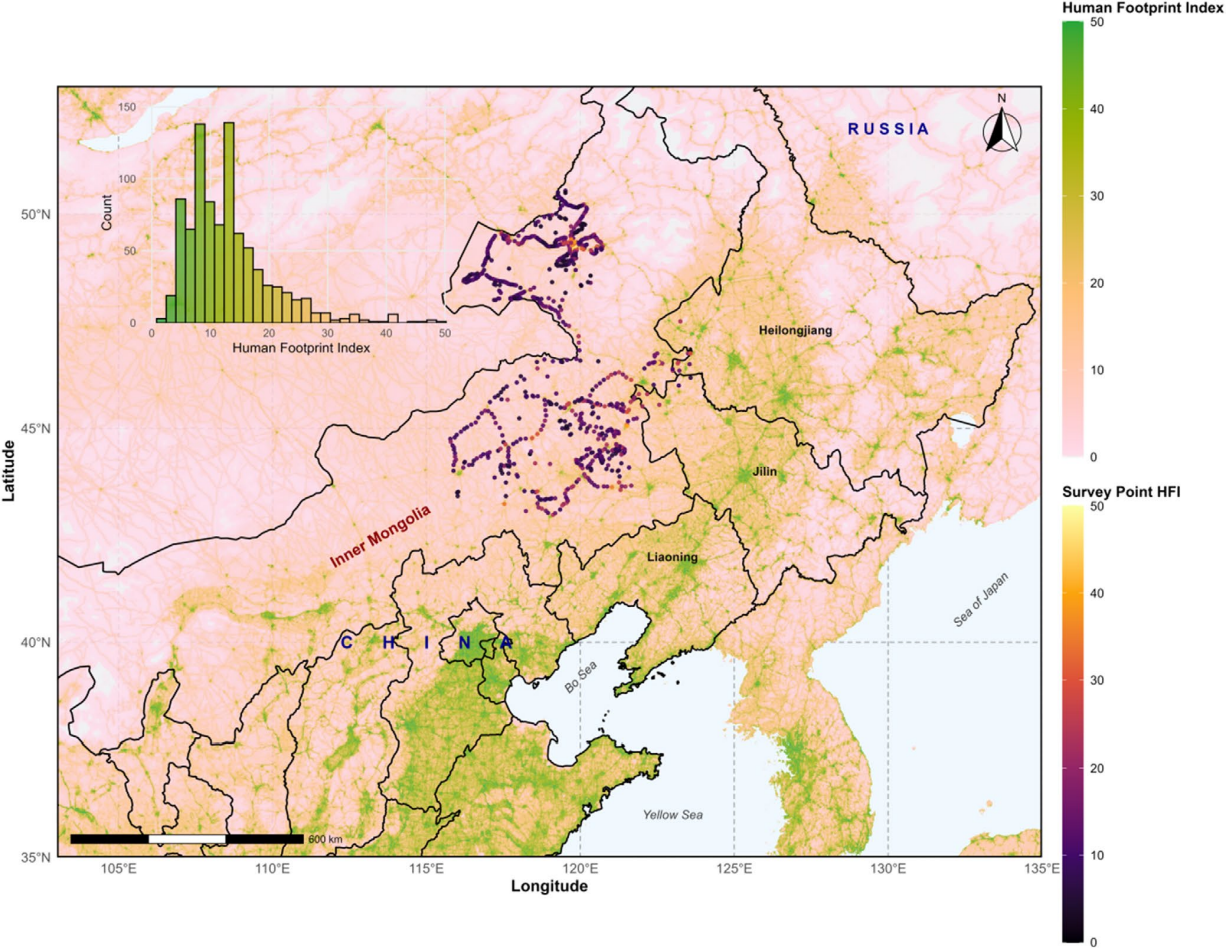
Spatial distribution of avian survey points across the study area in northeastern Inner Mongolia, China. The Human Footprint Index (HFP) at each point is depicted in the inset histogram, whereas background HFP values are visualized as a gradient.

The study was conducted in northeastern Inner Mongolia, known for its temperate climate and diverse vegetation zones, including coniferous and deciduous forests, meadow steppe, and typical steppe (Han et al. 2009; Wu et al. 2015). The region has a temperate climate, with cold winters and mild summers. The mean annual temperature is 6°C–7°C, and annual precipitation ranges from 300 to 400 mm (Wu et al. 2015). The study area spans a gradient from relatively undisturbed grasslands to landscapes heavily altered by human activity. Altered landscapes include croplands, bare lands (e.g., exposed soil, sand, or fallow fields), tree plantations (poplar and Sibirica species aged 3–10 years for timber and fruit production), and various human settlements (from small towns to cities with populations ranging from 10,000 to 138,000). This diverse range of environmental conditions makes the region an ideal location for examining the impacts of human‐induced disturbances on steppe bird communities. It provides a natural gradient of human impact to assess species‐specific responses and disturbance tolerance thresholds (Figure 1).

### 2.2 Bird Surveys and Human Footprints

We surveyed bird communities at 904 points using standardized point‐count methods (Bibby et al. 2000). These points were chosen for their accessibility and representativeness, ensuring coverage of areas with varying levels of human modification. To avoid biases from proximity to major disturbances, survey points were located at least 100 m from clear‐cuts, roads, or urban areas, and at least 2000 m apart to prevent double‐counting. Trained ornithologists conducted 10‐min counts, recording all birds seen or heard within a 100‐m radius. This duration was selected to optimize detection rates and minimize observer bias (Bibby et al. 2000). Surveys were conducted from 2020 to 2024, with each site visited twice (once before and once after mid‐May, separated by 4–6 weeks). Only birds actively foraging or displaying were included. Species were recorded as present if detected at least once during the two visits, with relative abundance indicated by the maximum count (Reif et al. 2010). Bird surveys were conducted in the early morning under calm weather conditions.

The 2022 Human Footprint Index (HFP) was used to assess human impact. The HFP is a metric that combines anthropogenic pressures from land use, population density, transportation, and urban development (Mu et al. 2022). The index ranges from 0 (low disturbance, e.g., pristine wilderness) to 50 (high disturbance, e.g., dense urban centers). To aid interpretation, HFP values below 10 typically represent low‐impact areas like remote grasslands; 10–30 indicate moderate disturbances such as scattered agriculture or light infrastructure; and above 30 signify high‐impact zones with intensive urbanization or farming (Venter et al. 2016; Mu et al. 2022). The HFP provides a spatially explicit measure of human disturbance. HFP values were extracted for each survey point using the ‘extract’ function in the R package raster (Hijmans 2025).

### 2.3 Statistical Analysis

We excluded raptors and wetland birds with large territories, as well as species detected at fewer than 10 sites, leaving 25 species for analysis. This approach prioritized well‐documented distributions, improving the accuracy of ecological assessments. We used Threshold Indicator Taxa Analysis (TITAN) to identify species‐ and community‐level thresholds along the HFP gradient (Baker and King 2010). TITAN uses indicator species analysis and change‐point analysis to detect the value along an environmental gradient where a taxon shows a significant, abrupt change in occurrence or abundance. It bootstraps the data to estimate the reliability and purity (consistency in response direction) of each taxon's threshold, classifying them as positive (z+) or negative (z−) responders. The community‐level threshold is identified as the point where the cumulative response of all declining (sum z−) or increasing (sum z+) species is maximized, representing the point of greatest compositional turnover. We used 500 bootstrap replicates (default setting) to estimate purity (consistency of taxon response direction) and reliability (consistency across bootstrap cycles), which helps classify species as strong decreasing (“z−”) or increasing (“z+”) indicators at their identified thresholds. TITAN also identifies community‐level thresholds by pinpointing the HFP value where the cumulative response of all declining species (sum z−) or all increasing species (sum z+) is maximized. It provides metrics, such as change points for negative and positive responders, along with quantiles (0.05, 0.10, 0.50, 0.90, 0.95) to assess variability. This reveals the HFP level at which the most significant compositional turnover occurs, marking a substantial point of ecological change for the avian community. The analysis was performed using the “TITAN2” package in R (Baker and King 2013).

To model species richness and Shannon diversity in relation to HFP, we used piecewise regression (Muggeo 2003; Toms and Lesperance 2003). This method identifies breakpoints (thresholds) where the slope of the relationship changes significantly, enabling the detection of potential non‐linear patterns. Evidence for IDH would show a significant increase in diversity up to an intermediate HFP breakpoint, followed by a significant decrease beyond that point. It would quantify the “optimal” HFP, as well as the rates of increase and decrease. The analysis was performed using the segmented package in R, with confidence intervals derived from 1000 permutations (Muggeo 2008). Although TITAN identifies thresholds on the basis of compositional turnover of individual species, piecewise regression identifies thresholds on the basis of aggregate community metrics (richness and diversity). Both methods are widely applied in disturbance ecology; several studies employ both or similar approaches to validate thresholds. For example, in grazing‐induced changes (Li et al. 2017), species‐level responses (similar to TITAN) were tested with generalized linear, quadratic, and piecewise regressions to confirm true thresholds, showing that piecewise helps verify non‐linearity in aggregate functions. In water quality assessments (Sultana et al. 2020), TITAN was compared to generalized filtering (GF), but piecewise is noted as a robust alternative for single‐response thresholds. In practice, when applied to the same dataset, both methods output change points with quantiles or CIs, allowing for a direct comparison: if the community compositional threshold (from TITAN) occurs at a lower HFI value than the diversity peak (from piecewise regression), it indicates that sensitive species begin to be lost before overall diversity metrics decline. Finally, we compared the community threshold identified by TITAN with breakpoints detected by segmented regression. This comparison helps determine whether the point of maximum compositional change aligns with the peak or decline in overall diversity. All analyses were conducted using R version 4.4.3 (R Core Team 2025).

## 3 Results

**FIGURE 2:**
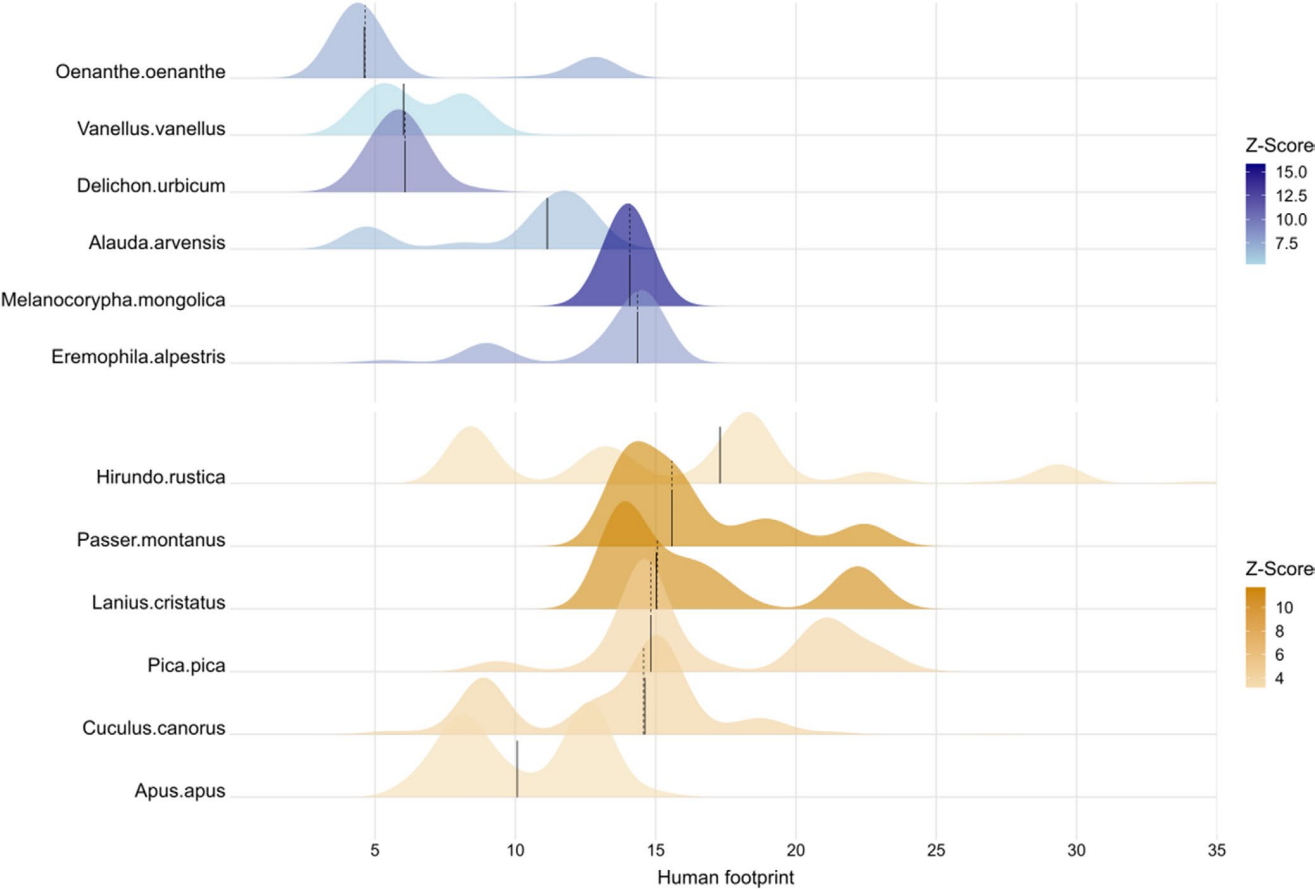
Ridgeplot of species‐specific abundance responses to the Human Footprint Index gradient. Only indicator species with high purity (≥ 0.95) and reliability (≥ 0.95) are shown. Peaks denote thresholds where species decline (left) or increase (right) in abundance. Z‐score is the IndVal z score derived from TITAN analysis.

The TITAN analysis revealed that species respond differently to the HFP gradient, with thresholds ranging from 3.37 (Rock Pigeon, *Columba livia* ) to 43.22 (Japanese Quail, *Coturnix japonica* and Hoopoe, *Upupa epops* ) (Table 1). The ridgeplot illustrates these responses, with species like Skylark and Mongolian Lark showing peak density around HFP = 15–20, with high Z‐scores (∼12.5–15.5, Figure 2). In contrast, species like Northern Wheatear ( *Oenanthe oenanthe* ) peak at lower HFP levels (around 5–10), indicating greater sensitivity to human impact (Figure 2). Species such as Skylark (threshold = 11.32, IndVal = 19.72) and Mongolian Lark (threshold = 14.14, IndVal = 29.49) had high indicator values, suggesting they are strong indicators of change at their respective thresholds (Table 1). Similarly, species with high purity and reliability (e.g., Northern Wheatear, threshold = 3.92, IndVal = 39.48) are particularly robust indicators (Table 1).

**TABLE 1:** Species‐specific and community‐level thresholds for 25 steppe birds along the Human Footprint Index (HFP) gradient, derived from Threshold Indicator Taxa Analysis (TITAN). Top: Species‐specific thresholds (zenv.cp), indicator values (IndVal), response direction (maxgrp: 1 = decline, 2 = increase), purity/reliability metrics, and filtering status (filter: 0 = excluded, 1/2 = included with maxgrp). Bottom: Community change points (cp) and quantiles (0.05–0.95) for sumz (unfiltered) and fsumz (filtered) scores.

	Species	zenv.cp	IndVal	maxgrp	Purity	Reliability	Filter
Species‐level threshold	*Alauda arvensis*	11.32	19.72	1	1.000	1.000	1
	*Anthus richardi*	12.73	8.8	1	0.978	0.934	0
	*Apus apus*	12.69	4.07	2	0.998	0.968	2
	*Columba livia*	3.37	17.99	1	0.422	0.822	0
	*Corvus corone*	5.23	6.77	1	0.620	0.924	0
	*Coturnix japonica*	43.22	18.55	2	0.458	0.916	0
	*Cuculus canorus*	15.13	9.85	2	1.000	0.988	2
	*Delichon urbicum*	6.11	10.2	1	0.964	1.000	1
	*Emberiza cioides*	22.61	4.61	2	0.908	0.874	0
	*Emberiza jankowskii*	6.80	3.04	2	0.756	0.698	0
	*Eremophila alpestris*	14.47	9.7	1	1.000	1.000	1
	*Hirundo rustica*	18.29	15.87	2	0.988	0.974	2
	*Lanius cristatus*	13.58	14.1	2	1.000	1.000	2
	*Melanocorypha mongolica*	14.14	29.49	1	1.000	1.000	1
	*Motacilla alba*	32.89	12.88	2	0.910	0.964	0
	*Motacilla flava*	12.43	1.44	1	0.776	0.604	0
	*Oenanthe isabellina*	13.56	4.08	1	0.944	0.914	0
	*Oenanthe oenanthe*	3.92	39.48	1	1.000	1.000	1
	*Passer montanus*	14.11	37.52	2	1.000	1.000	2
	*Phasianus colchicus*	21.10	4.87	2	0.970	0.938	0
	*Pica pica*	14.79	14.35	2	0.982	0.984	2
	*Streptopelia decaocto*	14.27	6.26	2	0.874	0.996	0
	*Upupa epops*	43.22	33.62	2	0.840	0.834	0
	*Vanellus cinereus*	7.76	2.13	1	0.708	0.744	0
	*Vanellus vanellus*	6.03	9.02	1	0.978	0.988	1
	**Community**	**cp**	**0.05**	**0.10**	**0.50**	**0.90**	**0.95**
Community‐level threshold	sumz−	13.65	4.90	5.02	13.15	14.10	14.16
	sumz+	14.24	13.65	13.85	14.27	18.22	19.98
	fsumz−	13.37	5.01	5.28	13.19	14.24	14.42
	fsumz+	14.24	13.31	13.60	14.46	16.69	18.70

**FIGURE 3:**
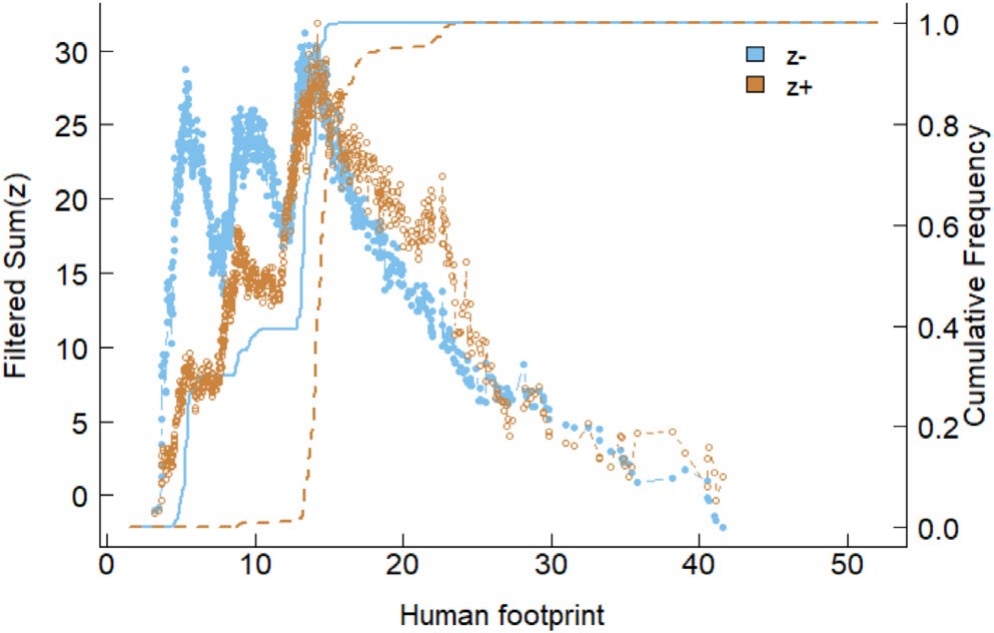
Community‐level threshold response to the Human Footprint Index gradient. The filtered sum of z‐scores (fsumz) peaks at HFP = 13.65 (negative response) and 14.24 (positive response).

The community threshold was observed at HFP ≈ 14 (Table 1). Specifically, the change point for negative responders (sum z−) was 13.65 and for positive responders (sum z+) was 14.24 (Table 1). The community threshold plot visually confirms this, showing a peak in the filtered sum of Z‐scores around HFP = 10–15, indicating a critical threshold where community composition shifts (Figure 3). The plot shows blue points (z−) and orange points (z+), with trend lines converging around HFP = 20 (Figure 3), suggesting stabilization after the threshold.

**FIGURE 4:**
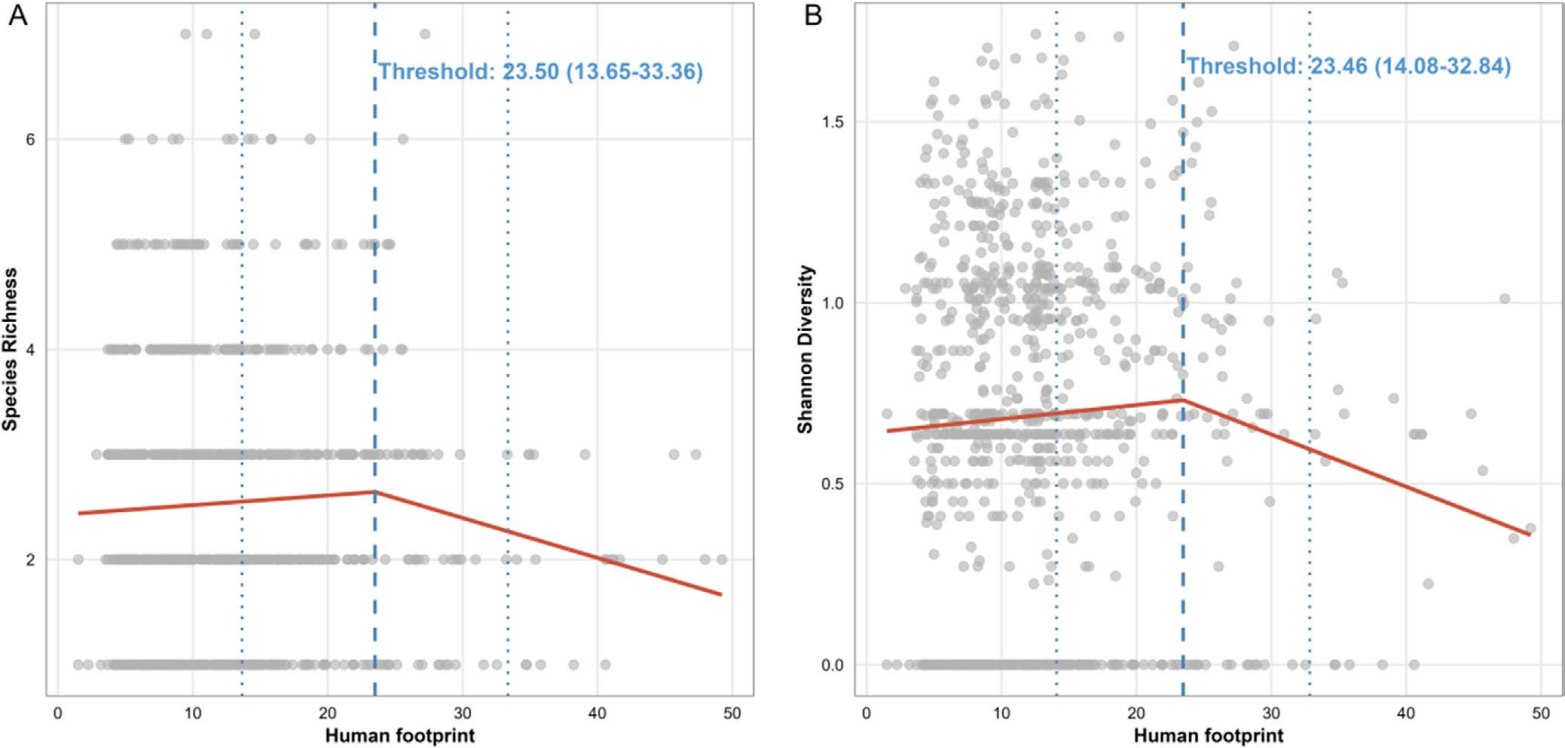
Piecewise regression models for (A) species richness and (B) Shannon diversity across the Human Footprint Index (HFP) gradient. Red lines indicate the fitted linear model trends, and vertical dashed lines indicate thresholds (breakpoints) and 95% CI.

Piecewise regression identified thresholds for species richness at HFP = 23.50 (CI: 13.65–33.36) and for Shannon diversity at HFP = 23.46 (CI: 14.08–32.84, Figure 4). Below these thresholds, both metrics either increased slightly or remained stable as HFP increased (slopes: richness = 0.009, diversity = 0.004). However, above these thresholds, both metrics declined (slopes: richness = −0.038, diversity = −0.014, Table S1), indicating that high HFP negatively affects biodiversity.

## 4 Discussion

Variation in species‐specific thresholds along the human footprint gradient highlights the differential sensitivity of avian species to anthropogenic disturbance. This pattern aligns with established ecological theory, which predicts that species with varying life‐history traits, habitat specializations, and behavioral plasticity will exhibit distinct tolerance thresholds to human modification (Tucker et al. 2018; Etard and Newbold 2024). For instance, the low threshold observed in species such as the Northern Wheatear suggests a high sensitivity, likely due to specialized habitat requirements or behaviors that help them avoid disturbances (Han et al. 2025), similar to other ground‐nesting or open‐habitat specialists (Guerrero et al. 2012; Han et al. 2020). In contrast, species like the Barn Swallow ( *Hirundo rustica* ) and Eurasian Tree Sparrow ( *Passer montanus* ) exhibit higher thresholds, reflecting their greater adaptability to human‐modified environments and reliance on human infrastructure, as documented in other regions (Grüebler et al. 2010; Ambrosini et al. 2012; Ahmad et al. 2025).

High indicator values and associated purity/reliability metrics for species like the Skylark and Mongolian Lark establish them as robust ecological indicators of change at their specific HFP thresholds (approximately 11–14). Their peak densities and high Z‐scores within the HFP 15–20 range highlight a critical transition zone where these species are most responsive (Figure 2). The strength of these indicator species, particularly those with high purity like Northern Wheatear, provides valuable tools for monitoring ecosystem integrity, as their presence and abundance offer clear signals of specific levels of anthropogenic pressure (Fleishman et al. 2005; Morelli et al. 2017; Han et al. 2023).

TITAN analysis also revealed a significant community‐level compositional threshold at HFP around 14 (sum z− = 13.65, sum z+ = 14.24, Table 1). This convergence point, visually confirmed by the peak in the filtered sum of Z‐scores, represents a tipping point where the assemblage structure undergoes a fundamental reorganization. This HFI value (≈14) corresponds to a landscape of low‐intensity agriculture and rangelands (Venter et al. 2016), indicating that even these moderate human disturbances can alter the structure of bird communities, potentially leading to the loss of sensitive species and the dominance of more tolerant or opportunistic species (Devictor et al. 2008; Neate‐Clegg et al. 2023). The stabilization of trend lines around HFP of 20 suggests a transition toward a novel, disturbance‐tolerant community state.

Importantly, the shift in community composition occurs before noticeable changes in biodiversity metrics. Piecewise regression identified a distinct threshold for species richness and Shannon diversity at HFP ≈ 23.5, much higher than the community compositional threshold (HFP ≈ 14). This indicates that species composition and relative abundances start changing at moderate HFP levels, but the total number of species and diversity metrics remain relatively stable—or even increase slightly—until higher anthropogenic pressure is reached. This pattern is consistent with the phenomenon of biotic homogenization, where sensitive specialists are replaced by widespread generalists, often preceding measurable declines in alpha diversity in human‐modified landscapes (Olden and Rooney 2006; Oliver et al. 2015). This lag between compositional change and diversity loss is a potential manifestation of extinction debt (Hylander and Ehrlén 2013), where species persist for a time in suboptimal habitats but are committed to future local extinction. Such a pattern could serve as an early warning signal, allowing for proactive conservation measures before diversity declines become apparent (Scheffer et al. 2009).

Species richness and Shannon diversity peak at intermediate HFP levels (≈23.5, Table S1, Figure 4), which provides support for the Intermediate Disturbance Hypothesis in this system. The peak occurs at a “low‐intermediate” level of human footprint, corresponding to mixed agricultural and semi‐natural landscapes, which may initially support higher diversity by creating diverse habitats or resources. However, this support is qualified: the lower community threshold (~14) indicates an early, rather than truly intermediate, shift, potentially representing a low‐to‐moderate disturbance level where sensitive species are already lost. This aligns with critiques that anthropogenic disturbances may not fully mimic natural ones, often leading to net biodiversity loss (Fox 2013; Murphy and Romanuk 2012). Although intermediate HFI may temporarily elevate diversity through entry of generalists, this does not imply human disturbance is inherently favorable for biodiversity; rather, it highlights the need for managed reconciliation in anthropized landscapes (Rosenzweig 2003; Hobbs et al. 2006). Over time, persistent disturbance might favor generalists, reducing functional diversity and incurring extinction debts.

Additionally, the negative slopes for both metrics above their respective HFP thresholds confirm the detrimental impact of high anthropogenic pressure on overall biodiversity (Di Marco et al. 2018; Williams et al. 2020; Marjakangas et al. 2024). The transition from slight stability or increase below the threshold to a decline above it highlights a non‐linear response, consistent with ecological threshold theory (Huggett 2005; Salgueiro et al. 2018; Dakos et al. 2019). This decline likely reflects the cumulative loss of habitat specialists, increased competition from synanthropic species, and other stressors linked to intensive human land use that exceed the resilience of many species (Isbell et al. 2017; Newbold et al. 2020). The ecological consequences of these shifts are significant. The loss of specialized species, particularly ground‐nesting insectivores like the Northern Wheatear and various larks, at low HFI levels could disrupt key ecosystem functions such as pest control and nutrient cycling (Whelan et al. 2015). Relying solely on richness/diversity thresholds might overestimate sustainable HFI levels, missing early composition shifts that erode functional roles. For instance, homogenized communities (sensu Olden and Rooney 2006, as loss of unique species assemblages) may appear diverse but lack keystone species, leading to cascading effects on pollination and pest control. Our results show that community composition is a more sensitive indicator of environmental stress.

## 5 Conservation Implications

These findings have several important conservation implications. First, maintaining HFP levels below the community threshold (HFP < 14) could help preserve original community composition. Keeping HFP levels below 23.5 might still support relatively high biodiversity, albeit with a different community structure. However, exceeding HFP = 23.5 could lead to significant biodiversity loss. Land‐use planning and protected area management should prioritize maintaining HFP below 14 in core habitats for sensitive species, and below 23.5 in broader landscapes to prevent biodiversity loss (Kehoe et al. 2015; Perring et al. 2015). Second, species‐specific thresholds can inform targeted conservation actions. For example, species like the Northern Wheatear, which have low thresholds and high indicator values, may be particularly vulnerable and thus warrant protection. Monitoring these indicator species can provide valuable insights into the health of bird communities under varying levels of human impacts. Third, the observed peak in diversity at intermediate HFI should not be misinterpreted as a justification for promoting disturbance. Rather, it underscores the potential for “reconciliation ecology” in moderately used landscapes (Rosenzweig 2003). Conservation efforts should focus on managing these human‐dominated matrices to maintain habitat heterogeneity and resources for both disturbance‐tolerant and more sensitive species, preventing further simplification toward a depauperate state dominated by a few synanthropic species (Hobbs et al. 2006).

## 6 Conclusion

The findings underscore the importance of considering both community‐level and species‐specific responses when assessing the impact of human activities on steppe birds. Moreover, support for the Intermediate Disturbance Hypothesis in this context highlights the complex relationship between human impact and overall diversity, suggesting that conservation efforts should aim to maintain habitats across a range of disturbance levels to maximize biodiversity. Our results confirm IDH for overall diversity metrics but crucially show that community composition is negatively impacted at lower disturbance levels. This means the “optimal” state for diversity is already a degraded state from a compositional and potentially functional perspective. By identifying early warning signals through community thresholds, this study provides a framework for proactive conservation strategies. Future research should explore the mechanisms driving these thresholds, such as changes in habitat quality, food availability, or predation pressure. Longitudinal studies could track bird community responses to changes in HFP over time. Furthermore, incorporating other taxonomic groups or using more detailed spatial data could provide a more comprehensive understanding of the effects of human footprints on biodiversity.

## Author Contributions

**Xi Yang:** formal analysis (equal), investigation (equal), methodology (equal), visualization (equal), writing – original draft (equal), writing – review and editing (equal). **Lishi Zhang:** investigation (equal), methodology (equal), resources (equal), writing – review and editing (equal). **Piotr Tryjanowski:** methodology (equal), resources (equal), validation (equal), writing – review and editing (equal). **Frédéric Jiguet:** conceptualization (equal), methodology (equal), resources (equal), writing – review and editing (equal). **Zheng Han:** conceptualization (equal), formal analysis (equal), funding acquisition (equal), investigation (equal), supervision (equal), visualization (equal), writing – original draft (equal), writing – review and editing (equal). **Haitao Wang:** conceptualization (equal), investigation (equal), methodology (equal), visualization (equal), writing – original draft (equal), writing – review and editing (equal).

## Funding

This work is supported by the National Natural Science Foundation of China (No. 32201304) and the Fundamental Research Funds for the Central Universities (No. 2412022QD026).

## Ethics Statement

The authors have nothing to report.

## Conflicts of Interest

The authors declare no conflicts of interest.

## Supporting information


**Table S1:** Threshold values and piecewise regression slopes for species richness and Shannon diversity along the Human Footprint Index (HFP) gradient. Thresholds (with 95% CIs) and slopes below/above thresholds are also shown.

**Data S1:** ece372683‐sup‐0002‐Supinfo.csv.

## Data Availability

Data are provided as Supporting Information.
