# Supplementary material for: Threshold Responses of Bird Communities to Human Footprint: Testing the Intermediate Disturbance Hypothesis and Implications for Biodiversity Conservation
Source: Ecol Evol. 2025 Dec 16;15(12):e72683. doi: 10.1002/ece3.72683 (PMC12706524; doi:10.1002/ece3.72683)
Supplement: Supplementary file 1 — Table S1: Threshold values and piecewise regression slopes for species richness and Shannon diversity along the Human Footprint Index (HFP) gradient. Thresholds (with 95% CIs) and slopes below/above thresholds are also shown. [file ECE3-15-e72683-s001.docx]

Table S1. Threshold values and piecewise regression slopes for species richness and Shannon diversity along the Human Footprint Index (HFP) gradient. Thresholds (with 95% CIs) and slopes below/above thresholds are also shown.

| Metric | Threshold | CI Lower | CI Upper | Slope below | Slope above |
| --- | --- | --- | --- | --- | --- |
| Species Richness | 23.504 | 13.649 | 33.360 | 0.009 | -0.038 |
| Shannon Diversity | 23.461 | 14.079 | 32.844 | 0.004 | -0.014 |
